# Exploiting the Nucleotide Substrate Specificity of Repair DNA Polymerases To Develop Novel Anticancer Agents

**DOI:** 10.3390/molecules16097994

**Published:** 2011-09-16

**Authors:** Emmanuele Crespan, Anna Garbelli, Alessandra Amoroso, Giovanni Maga

**Affiliations:** DNA Enzymology & Molecular Virology, Insititute of Molecular Genetics IGM-CNR, via Abbiategrasso 207, I-27100 Pavia, Italy; Email: emmanuelecrespan@gmail.com (E.C.); agarbelli@gmail.com (A.G.); ale19812002@yahoo.it (A.A.)

**Keywords:** DNA repair, cancer, DNA polymerase, nucleoside analogs

## Abstract

The genome is constantly exposed to mutations that can originate during replication or as a result of the action of both endogenous and/or exogenous damaging agents [such as reactive oxygen species (ROS), UV light, genotoxic environmental compounds, *etc*.]. Cells have developed a set of specialized mechanisms to counteract this mutational burden. Many cancer cells have defects in one or more DNA repair pathways, hence they rely on a narrower set of specialized DNA repair mechanisms than normal cells. Inhibiting one of these pathways in the context of an already DNA repair-deficient genetic background, will be more toxic to cancer cells than to normal cells, a concept recently exploited in cancer chemotherapy by the synthetic lethality approach. Essential to all DNA repair pathways are the DNA pols. Thus, these enzymes are being regarded as attractive targets for the development of specific inhibitors of DNA repair in cancer cells. In this review we examine the current state-of-the-art in the development of nucleotide analogs as inhibitors of repair DNA polymerases.

## 1. DNA Damage Response

The genome is constantly exposed to mutations. Mutations in DNA can originate during replication as a result of “copying mistakes”, or they can derive from the action of both endogenous and exogenous damaging agents [such as reactive oxygen species (ROS), UV light, genotoxic environmental compounds, *etc*.], which alter the chemical structure of DNA bases resulting in an increased error rate by replicative DNA polymerases (pols) or single-strand DNA breaks (SSBs) and/or double strand breaks (DSBs). For the normal cell survival in mammals, more than 150 different proteins involved in the response to DNA damage are necessary. These proteins are responsible for ensuring the DNA integrity and the stalling of the cell cycle to allow repair to occur [1,2]. In fact, in the absence of DNA repair, the cell could not bypass the various lesions that may be generated during the cell cycle.

Eukaryotic cells have developed a complex series of processes to deal with DNA lesions, collectively called the DNA damage response (DDR) pathway. DDR is required for avoiding DNA damage caused by replication errors and preventing transmission of mutations to daughter cells. When DNA is affected by some injury, cell cycle checkpoints are activated to block normal cell cycle progression and to promote damage sanitation by specific DNA repair mechanisms. 

DDR activation requires the presence of two kinases that start up two distinct pathways depending on the DNA lesion. The ataxia telangiectasia mutated (ATM) kinase is involved in the response to the DSBs caused by ionizing radiation (IR), while when the lesion induces stalling in DNA replication forks, the ATM-Rad3-related (ATR) kinase is activated. These two kinases start a cascade of events that regulate cell cycle checkpoints, retrieve the proteins required for the different DNA repair pathways and, eventually, trigger apoptosis when the damage is unrepairable.

### 1.1. Defective DDR in Cancer

Alterations in DDR pathways are often associated with genetic mutations, gene amplification and chromosomal aberrations, that cause genetic instability syndromes and tumorigenesis. Among the principal human genetic syndromes associated with DDR genes/proteins mutations, there is Ataxia Telangiectasia (AT), which is caused by an ATM mutation and is characterized by the development of cancers and neurodegenerative diseases [3]. Another syndrome, Fanconi’s anemia, is caused by mutations in factors involved in repair of DNA crosslinks, and predisposes individuals to myeloid leukemias [4]. Bloom and Werner syndromes are due to defects in the BLM and WRN DNA helicases and are characterized by photosensitivity, growth deficiency, variable degrees of immunodeficiency and increased susceptibility to neoplasms. Xeroderma Pigmentosum (XP), Cockayne syndrome (CS) and Trichothiodystrophy (TTD), are disorders characterized by defects in the nucleotide excision repair (NER) pathway. Patients are characterized by photosensitivity, pigmentation changes, premature skin aging, and malignant tumor development [5]. The Li-Fraumeni syndrome is consequent to mutations in the tumor suppressor genes TP53 and *CHK2*, that are involved in DDR and causes several type of cancers such as breast cancer, osteosarcoma, cancer of soft tissue, brain tumors, leukemias and adrenocortical carcinoma [6]. Finally, many tumors also correlate with defects in DDR factors, for example mutations of either BRCA1 and BRCA2 genes predispose to breast and ovarian cancer [7,8]. Often, cancer cells compensate the presence of loss-of-function mutations in DDR, by acquiring gain-of-function phenotypes in other repair pathways, for example overexpressing certain DNA repair proteins. Thus, tumor cells are able to survive even with a defective DDR, but are dependent on fewer DNA repair pathways than normal cells. Thus, by studying the DDR network and understanding how its deficiency is implicated in cancer, it will be possible develop drugs that can specifically target DDR-deficient cancer for cancer treatment. The identification of relevant molecular targets in cancer-specific DDR, has provided, in recent years, new avenues for the treatment of different types of tumors.

### 1.2. Targeting DDR in Cancer Treatment: The “Synthetic Lethality Model”

The traditional anticancer chemotherapeutic approach has aimed to strike proliferating cells. Such a system, however, is not always effective for two main reasons: firstly, not all cancer cells have a high proliferative index, secondly, highly proliferating normal cells, such as in bone marrow, can also be damaged by these therapies. The consequences are a poor quality of life due to toxicity and a predisposition to develop cancers again due to incomplete cancer growth suppression. The development of new generation anticancer therapies is aimed to the selective targeting of cancer cells without affecting normal cells.

The “synthetic lethality” concept, delineated through genetic studies in *Drosophila* [9,10], described the conditions in which the loss of one gene function is tolerated by over-reliance on another gene in a redundant pathway. Ablating this second gene function results in cell lethality.

This kind of principle has been exploited to produce an innovative anticancer approach, in fact the loss of tumor suppressor genes is a common event in cancer onset and this property inspired Hartwell and colleagues [11]. They showed that blocking the gene product required to compensate for the tumor suppressor loss, could kill cancer cells. This idea is also supported by the fact that often in tumors both copies of tumor suppressor genes are deleted, but a normal copy is present in healthy tissue, so “synthetic lethality” would be specific to the tumor cells [12,13].

Presently, the “synthetic lethality” approach has been successfully applied to the inhibition of poly(ADP-ribose) polymerase (PARP-1), which is involved in the identification of damages deriving from reactive oxygen species [14]. Recently, some small molecules based on nicotinamide analogs have been reported to function as inhibitors of PARP-1 [15,16]. An interesting result was obtained using PARP-1 inhibitors against BRCA1 and BRCA2 deficient tumor cells, in which killing was specifically directed again these cells with minimal effects on wild-type cells [17,18]. BRCA1 and BRCA2 proteins are involved in repair of DNA damage through the HR pathways and cells defective in these two proteins are unable to solve replication forks stalling caused by agents that produce interstrand crosslinks. The alternative pathway necessary to repair DSBs is NHEJ or a single-strand annealing (SSA) process that requires the intervention of the poly (ADP-ribose) polymerase PARP. If PARP activity is lost by using specific inhibitors, the formation of DNA lesions increases and, when this event is contemporary with deficiency of BRCA1 or BRCA2 proteins, a “synthetic lethality” situation occurs for the cancer cells [7]. Since BRCA1 or BRCA2 are notoriously inactivated in breast and ovarian cancer, the strategy described above may be considered an effective approach to hit cancer cells in a selective manner.

These studies provided the “proof-of-principle” for the synthetic lethality approach. In principle, any protein essential in DDR can be exploited in this context. One class of enzymes that might be particularly relevant for novel anticancer therapies are the DNA pols.

## 2. DNA Polymerases as Anticancer Drug Targets

There are multiple mechanisms for repairing the distinct DNA lesions deriving from different sources. Repair pathways are classically divided into nucleotide excision repair (NER), mismatch repair (MMR), base excision repair (BER) and DNA double strand break repair (DSBR) that includes homologous recombination (HR) and non-homologous end joining (NHEJ). There is also a pathway called translesion synthesis (TLS), that is an ubiquitous mechanism that support DNA synthesis past lesions that cannot be negotiated by the high-fidelity replicative DNA pols.

**Table 1:** Specialized DNA pols and their involvement in specific DNA repair pathways.

DNA repair pathways	DNA lesions recognized and removed	DNA polymerases involved	Related diseases
**NUCLEOTIDE EXCISION REPAIR (NER)**	Bulky lesions: *thymidine dimers*,* pyrimidine dimers*,* single-strand breaks*	pol α, pol β, pol δ, pol ε, pol κ, pol η	Xeroderma Pigmentosum (XP), Cockayne Syndrome (CS), trichothiodystrophy
**MISMATCH REPAIR (MMR)**	Base-base mismatches	pol β, pol δ	Hereditary nonpolyposis colorectal cancer (HNPCC), sporadic cancer (colorectal, gastric, endometrial, cervical, ovarian, breast, lung, bladder), gliomas, leukemia and lymphomas
**BASE EXCISION REPAIR (BER)**	Non-bulky lesions: *base modifications by alkylation and oxidation*, *single strand breaks (SSBs)*	pol β, pol λ, pol δ, pol ε	Solid tumors, chronic myeloid leukemia
**HOMOLOGOUS RECOMBINATION (HR)**	DNA gaps, DNA double strand breaks (DSBs), DNA interstrand crosslinks	pol δ, pol ε	Breast cancer, ovarian cancer, Fanconi anemia
**NON-HOMOLOGOUS ** **END JOINING (NHEJ)**	Double strand breaks (DSBs)	pol μ, pol λ, Terminal transferase (TdT), pol η	Leukemias
**TRANSLESION SYNTESIS (TLS)**	Abasic sites, bulky DNA template adducts, thymidine-thymidine or cyclobutane-pyrimidine dimers, cis-platinum adducts.	TLS polymerases η, ι, κ, ζ, Rev1	Xeroderma pigmentosum-variant (XPV)

These pathways have different substrate specificities and modes of action, however all of them require factors able to replace the lost or damaged DNA sequence with “original” or “correct” copies, usually derived from the unaltered complementary DNA strand. For this reason, DNA pols are the key players in DNA repair [19]. In fact, DNA pols are the only biological macromolecules able to duplicate the genetic information stored in the DNA, hence they are necessary during both DNA replication and repair. In each DNA repair pathways one or more specific DNA pols are required depending on damage kind, cellular cycle phase, DNA repair reaction and tissue specificity. The multiple DNA repair pathways in the cell are specialized in repairing specific DNA lesions by using different DNA pols as summarized in Table 1.

**Figure 1:**
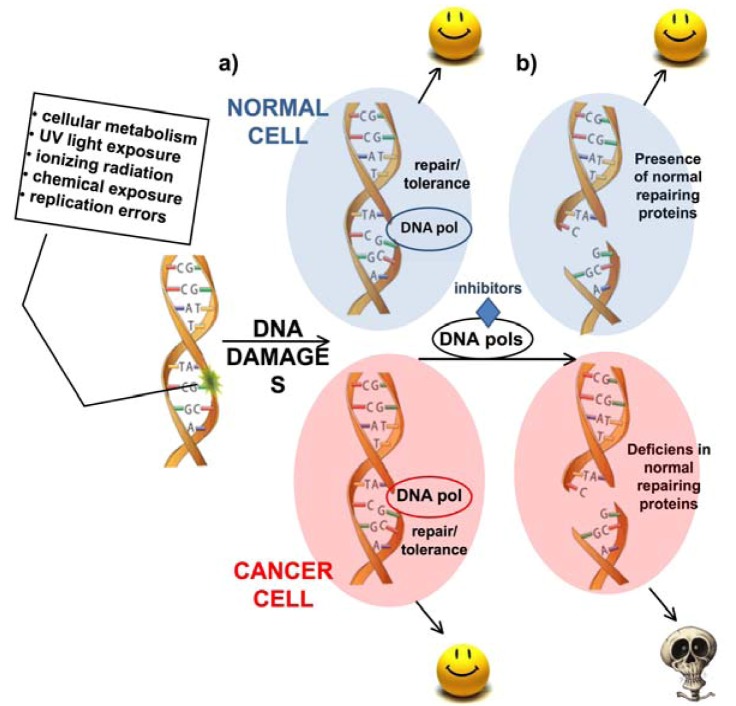
The “synthetic lethality” system represents a new therapeutic approach for selectively killing of cancer cells. Often cancer cells are deficient in specific DNA repair pathways or factors. (**a**) When cells are affected by DNA damages, specific DNA pols are activated that allow both normal or cancer cells to survive. (**b**) In the presence of specific DNA pols inhibitors, cells are not able to repair a DNA damage, which often causes SSBs and DSBs. Normal cells could bypass this problem since they have specific pathways available, instead inhibition of repair DNA pols in cancer cells that are defective in DSBs repair will result in synthetic lethality.

Recently the concept of “synthetic lethality” was exploited by targeting DNA pols in the context of deficient mismatch repair (MMR) components. MMR is involved in detection and repair of DNA replication that occur during replications, such as polymerase errors. MMR is also involved in the repair of oxidative lesions [20,21]. Mammalian cells usually repair these lesions with a BER mechanism that involved primarily DNA pol β in the nucleus and DNA pol γ in mitochondria. However, recent evidence suggests that the essential MMR factors, MLH1 and MSH2, are also implicated in 8-oxoG repair, and defects in these two genes result in an accumulation of 8oxoG lesions [21]. The overwhelming majority of hereditary nonpolyposis colorectal cancer (HNPCC) are attributed to mutations in the MMR genes MSH2 and MLH1 [22]. MSH2 and MLH1 are essential for MMR pathway and while tumor cells can have a complete loss of MSH2 or MLH1 functions, normal cells conserve at least one functional allele. For these intrinsic characteristics and for their ability to interact with DNA pols, found through a synthetic lethal screening in yeast [23], these two MMR components was considered good model for a “synthetic lethality” approach using DNA pol specific inhibitors. Indeed, Ashworth and co-authors demonstrated that both DNA pol β and DNA pol γ inhibition led to the accumulation of 8-oxoG oxidative DNA lesions. In particular they observed that MSH2-deficiency was synthetically lethal with inhibition of DNA pol β, while MLH1-deficiency was synthetically lethal with DNA pol γ inhibition [24].

These results open the way for a new attractive “synthetic lethality” approach in which cancer cells could be selectively target without impairing normal cells, by specific compounds directed against repair DNA pols (Figure 1). Other targets for anticancer therapy besides DNA pol β would be DNA pol ι and DNA pol λ, since these DNA pols are found overexpressed in 30% and 25% of analyzed tumors of many types, respectively [25]. DNA pol β and ι exhibit a very error-prone bypass of oxidative lesions and high levels of these two DNA pols correlate with genomic instability, high frequency of mutations and tumor progression [26,27,28]. On the contrary, DNA pol λ is highly proficient in error-free bypass of these lesions [29]. The fact that both DNA pol β and ι exhibit a very error-prone bypass of oxidative lesions [30], suggests that the simultaneous induction of DNA pol λ might reduce the levels of oxidative stress during tumor growth and development. The idea that springs out is that overexpression or increased activity of specialized DNA pols could result in enhanced damage tolerance capability, allowing cancer cells to better tolerate the high environmental stress that results from increased replication rates and higher levels of oxidative damage. Here, we will focus on the main repair DNA pols β and λ as anticancer drug targets.

### 2.1. DNA Pol λ in Mammalian Cells

DNA pol λ, identified in 2000, is a member of the X-family polymerases [31]. The corresponding gene, localized on chromosome 10 in humans, consists of nine exons forming a total 8 Kb genomic sequence. The human enzyme, with a 67–70 kDa molecular weight, contains 515 amino acids. DNA pol λ is characterized by the presence of a BCRT domain, as well as by several proline/serine-rich domains; an additional 8 kDa domain is also present in the protein, showing 32–33% homology with the corresponding region of the DNA pol β, and it possesses deoxyribose-5'phosphate-lyasic activity; another catalytic domain at the C-terminus, conserved in all family members, is responsible for the polymerase activity of this enzyme. DNA pol λ is mainly expressed in the testes, ovaries, fetal liver and moderately in other tissues. The enzyme can be phosphorylated by several cyclin/CDK-complex kinases *in vitro* and *in vivo* in a cell cycle regulated manner. DNA pol λ exists, therefore, in two forms: the first, hypophosphorylated and mainly present in the S-phase of the cell cycle, and the second, hyperphosphorylated in transition from G2 to M phase [32]. Phosphorylation stabilizes DNA pol λ during both the S and G2 phases of the cell cycle, allowing the enzyme to act in numerous biochemical processes, such as NHEJ, BER and TLS [33,34,35]. Its fidelity is reduced in the presence of Mg^2+^ ions, but it proved to be 5–6 fold increased with Mn^2+^ compared to DNA pol β [36]. This enzyme showed an efficient ability to elongate the DNA from a RNA primer annealed to the double-stranded DNA [37,38]. The DNA pol λ is also characterized by a terminal transferase activity (TdT), the atypical tendency to add nucleotides in the absence of a strand: this reaction seems to occur only in the presence of Mn^2+^ as activator. DNA pol λ can substitute for DNA pol β vitro BER with a 25% efficiency [34]. Other studies have an important role for DNA pol λ, in the NHEJ repair of double-strand breaks [33]. Finally, DNA pol λ was shown to be important in carrying out the error-free translesion synthesis opposite to the 8-oxoG damage and its efficiency was increased by two auxiliary human proteins: PCNA and RP-A [29,39,40,41]. Pol λ is phosphorylated at four distinct sites by cyclin dependent kinase 2 (Cdk2). These modifications did not affect any biochemical properties of pol λ but prevented its ubiquitination and its subsequent degradation [42]. The stabilization of pol λ was shown to occur in the late S and G2 phase following oxidative stress, consistent with a possible role in the repair of A:8-oxo-G mispairs [42,43]. DNA pol λ levels were also shown to fluctuate during tumor development with a kinetic different from DNA pol β, suggesting non-overlapping roles of these two enzymes in tumorigenesis [41].

### 2.2. DNA Pol β in Mammalian Cells

DNA pol β is the major DNA pol implicated in BER. This enzyme belongs, together with DNA pols μ and λ, to the DNA pol family X. It is able to associate with other BER enzymes such as DNA ligase I, AP endonuclease and XRCC1-DNA ligase III [44]. Moreover, DNA pol β bypasses cisplatin and oxaliplatin adducts [45]. DNA pol β predominantly bypasses the lesion by insertion of a complementary nucleotide to an adjacent downstream template site. This kind of DNA synthesis by “skipping over” the lesion results in both deletion and base substitution errors [46]. Many studies showed that transcriptional and protein levels of DNA pol β are higher in cancer tissues, especially solid tumors (e.g., prostate, breast, colon, ovarian) as well as in chronic myeloid leukemia [47]. These studies suggested that regulation of DNA pol β expression may be essential *in vivo* since its up-regulation could contribute to enhance chromosome instability and tumorigenesis when overexpressed just by 2-fold in cells [48]. Moreover, IR treatments in cells overexpressing DNA pol β resulted in increasing apoptosis and in a hypermutator phenotype in surviving cells. According to its proposed role as a mutator enzyme, DNA pol β exhibits a very low fidelity in DNA synthesis reaction opposite both 8-oxo-G and 1,2-dihydro-2-oxoadenine (2-OH-A) lesions and its overexpression enhances the mutagenicity of oxidative damages and increases apoptosis [26]. In the same way, down regulation of DNA pol β leads to deficient error-free DNA repair. This is then compensated by more error-prone pathways (such as TLS or NHEJ) resulting in higher rate of mutagenesis and increased cancer susceptibility [49]. DNA pol β is targeted for destruction by the E3 ubiquitin ligase CHIP under normal conditions but, when DNA damage occurs, it undergoes stabilization to increase the cellular capacity to perform BER [50,51].These data indicate that DNA pol β act as a determinant factor in both cell death and genetic changes associated with cancer [27] and pose DNA pol β as an attractive target for cancer therapy.

## 3. Nucleoside Analogs as DNA Pol β and λ Inhibitors

As of today, several classes of DNA pol λ and β inhibitors have been developed, but they are mostly non-nucleosidic compounds of natural origin such as: polypeptides, fatty acids, triterpenoids, sulfolipids, polar lipids, secondary bile acids, phenalenone-derivatives, anacardic acid, harbinatic acid, flavanoid derivatives and pamoic acid, only few are active and specific enough to be considered as DNA pol β or λ potential drug candidates. 

Nucleotide analogs (NAs) are extensively studied DNA pols inhibitors. The structures of these compounds resemble those of natural nucleotides in which the base or the sugar moieties are modified. Due to their similarity to canonical nucleotides, these compounds can interact with the catalytic site of the pols, competing with their natural substrates. However, the modifications of chemical and structural features of the natural nucleotides, confer upon the nucleotide analogs the ability to decrease the overall efficiency of the polymerase reaction. Nucleotide analogs are important drugs used for the treatment of many viral infections such as those caused by hepatitis B and hepatitis C viruses, herpes simplex virus and human immunodeficiency virus. These compounds can be incorporated into a nascent DNA strand; however, since they usually lack the 3' hydroxyl group, they cannot be elongated, acting as chain terminators and thus determining an abortive DNA replication. NAs are ideal candidates as drugs able to inhibit human DNA pols; they can act as chain terminators, and so impeding the elongation of the DNA growing strand, or directly inhibiting the DNA pol by tightly binding the catalytic site of these enzymes, impeding the incorporation of the canonical nucleoside substrates. Indeed, human DNA pols can be susceptible to drug inhibition by NA developed as viral inhibitors. The most compelling realization of this fact came with the correlation between their inhibition and onset of side effects during the administration of antiviral drugs [52]. For instance, NA employed in the treatment of HIV, namely Nucleoside Reverse Transcriptase Inhibitors (NRTIs), are substrates of Reverse Transcriptase (RT) but can also affect human DNA pols like pol β, λ and especially pol γ. For instance, alovudine (FTL), which exhibit great activity against RT, also strongly inhibits DNA pol γ. (Ki_app_ = 31 nM) and, to a lower extent, DNA pol β (Ki_app_ = 2.8 μΜ) [53,54]. In order to design potent antiretroviral NAs exhibiting lower side effects, a number of studies were performed to establish a structure-function relationship between NRTIs and RT, as well as human pols. These studies, in turn, can now be taken as a valuable source of information for the design, this time, of specific inhibitors of human DNA pols.

### 3.1. Nucleotide Analogs Cytotoxicity and Metabolism

#### 3.1.1. Substrate Specificity of Human DNA Pols towards Different Nucleotide Analogs

Kinetic studies performed with the approved NRTIs lamivudine (3TC), zidovudine (AZT), tenofovir and emtricitabine, showed that, in addition to RT, these antiviral nucleotide analogs are substrates for human DNA pols. All natural nucleotides, however, were incorporated better than these analogs either by the translesion DNA pols η, ι, κand Rev1, or by repair DNA pol λ and β [55,56].

It was shown that the cytotoxicity of AZT and zalcitabine (2'-3'-dideoxycytidine, ddC) increases in cell lines in which DNA pol β was overexpressed [57]. In particular, treatment with ddC inhibited *in vitr*o and *in vivo* the proliferation of DNA pol β transfected B16 melanoma cells and its administration also increased specifically the survival of mice bearing DNA pol β overexpressing B16 melanoma. This effect was due to the action of the active metabolite triphosphate form of ddC, since ddCTP electrotransfered into DNA pol β overexpressing B16 melanoma led to an even stronger growth inhibition (IC_50_ three times lower in DNA pol β overexpressing cells than in control cells) [47]. Moreover, short-term cardiac side effects of ddC administration in HIV infected patients are not dependent on pol γ inhibition [58]. Altogether these data seems to indicate that ddC, within human DNA pols, is specific toward DNA pol β; by contrast, *in vitro* data obtained with purified proteins, show that ddC inhibited DNA pol γ 39-fold better than DNA pol β (Ki = 1.2 and 0.034 μM respectively) [59]. The reason of these conflicting findings remains unclear.

The NRTI 3TC side effects are mainly related to mitochondrial toxicity. Indeed 3TC is efficiently incorporated into a growing DNA strand by DNA pol γ and acts as chain terminator. When the D- and L-enantiomers of 3TC were compared as DNA pol γ inhibitors, the L-enantiomer of 3TC resulted less active than its D-enantiomer, with a IC_50_ of 43.8 μM. L-3TC also inhibited DNA pol β with lower potency with respect to the D-enantiomer, but it resulted however more potent (IC_50_ = 24.8 μM) than toward DNA pol γ [60]. The currently approved drug for HIV infections treatment (lamivudine) is indeed L-3TC. These studies indicated that this ability of DNA pol β to utilize L-enantiomers of NAs, could be relevant for the design of compounds that can be specifically incorporated into DNA by this enzyme, even though the L-configuration of the sugar is not enough to ensure selectivity (see below).

NAs have been also successfully used in the treatment of various tumors. Currently, eight nucleoside analogs have been approved by FDA for cancer treatment: mercaptopurine, thioguanine, fludarabine and cladribine (purines), cytarabine and gemcitabine (pyrimidines), fluorouracil and capecitabine (fluoropyrimidines). Recently, additional novel purine analogs have been advanced to clinical phase: clofarabine (CAFdA), nelarabine, immucillin H (BCX-1777, forodesine) and 8-chloroadenosine (8-Cl-Ado). As in the case of the antiviral NAs drugs, they are administered as prodrugs that are similarly metabolized by endogenous nucleoside kinases to their nucleotide forms within the cells. These NAs can act through different mechanisms including: (i) inhibition of *de novo* synthesis of nucleosides and nucleotides; (ii) inhibition of the DNA chain elongation or (iii) induction of single strand breaks after their incorporation into the DNA strand. Most of these drugs are poor substrates for repair DNA pols, but a few have been shown to act as inhibitors of DNA pol β and/or λ.

For instance, 2-chloro-(2-deoxyﬂuoro-β-D-arabinofuranosyl)adenine (clofarabine), a deoxyadenosine analog that show excellent activity against lymphoproliferative malignancies, is a potent inhibitor of ribonucleotide reductase as well as DNA pol α and DNA pol ε, while it show poor activity on repair DNA pols [61].

Different from clofarabine, the β-L-dioxolane cytidine, L-(-)-deoxy-3'-oxacytidineroxacitabine (troxacitabine), an L-enantiomer analog of dideoxycytidine (ddC) and the only L-enantiomer employed as an anticancer drug, does not inhibit ribonucleotide reductase and acts as a chain terminator once incorporated into the DNA. It shows cytotoxic activity against a broad range of human tumor cell lines and human tumor xenograft models and is under investigation in clinical trial on haematological tumors and solid cancers [61]. Troxacitabine is efficiently incorporated by cellular DNA pols including pol α, pol ε, β and pol δ. In particular, it is less efficiently incorporated by DNA pol δ, with a selectivity *versus* the natural substrate dCTP (Km troxacitabine/Km dCTP) of 8.8, while DNA pol γ and β show the best incorporation efficiencies, with selectivities *versus* dCTP of 0.8 and 1.5, respectively.

1-β-D-Arabinofuranosylcytosine (cytarabine, araC) is successfully used in the treatment of acute myeloid leukemia and hematological malignancies [62], while 2',2'-difluoro-2'-deoxycytidine (gemcitabine, dFdC) is used as a single agent or in combination chemotherapy in the treatment of several cancers like non-small cell lung [63], pancreatic [64,65], ovarian [66], and breast cancer [67,68], as well as in the treatment of hematological malignancies [69]. Both these drugs are analogs of 2'-deoxycytidine (dC); after being incorporated into a DNA strand, they act as inhibitors of DNA synthesis of DNA pols α and ε, causing the stall of the replication forks. DNA repair pols also seem to play an important role in the cytotoxicity of araC and dFdC. In particular the major route of incorporation of araC into the genome is through DNA synthesis following DNA repair [70], while dFdC results less toxic to BER-deficient cells [71]. In a recent work [72], it was demonstrated that pol β is able to incorporate araCTP and dFdCTP into a gapped DNA template with catalytic efficiencies close to the natural substrate dCTP (8.8- and 47- fold lower than dCTP incorporation for araC and dFdC respectively). Moreover, in contrast to efficient insertion of araCTP, pol β is extremely slow at extension of an araC-terminated DNA primer [73], suggesting that the strong chain termination activity of araC could be partially explained by this pol behaviour. Similarly to DNA pol β, DNA pol λ is able to incorporate araCTP with an efficiency close to dCTP (kcat/Km 14-fold lower for araCTP incorporation than dCTP) [74], while dFdCTP is incorporated in a gapped DNA with an efficiency 146-fold lower than the natural substrate dCTP. These differences can be explained by the conformation of the ribose moieties of araCTP and dFdCTP within the active site of DNA pol λ bound to a gapped DNA: the first one is similar to that of normal dCTP while the conformation of dFdCTP is significantly different. The ability of DNA pol λ to incorporate araC into the DNA is consistent with the possibility that this enzyme contributes to the cytotoxic effect of AraC.

**Figure 2:**
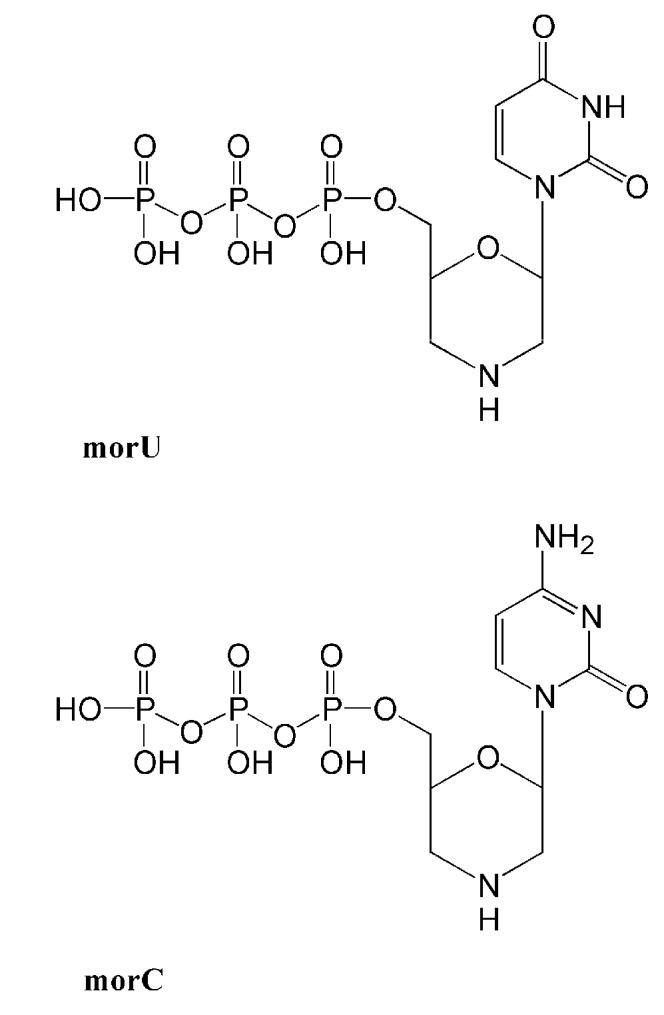
Structures of morpholinonucleoside triphosphates.

A property of DNA pol β that emerges from data obtained studying antiviral and anticancer NAs, is its ability to utilize NAs containing a modified ribose residue. This feature could be relevant for the design of DNA pol β selective NAs inhibitors. Recently, it was found that morpholinonucleoside triphosphates (dNTP analogs containing the morpholino residue instead of deoxyribose) can be good substrates both for HIV RT and DNA pol β. In particular, DNA pol β showed high efficiencies of incorporation of compound morC and morU (Figure 2) with Km values of 2.5 and 0.28 μM respectively [75]. It has to be noted that this activity was observed employing Mn^2+^ as metal co-activator while it is thought that DNA pol β utilized Mg^2+^* in vivo* as cofactor; on the other hand DNA pol λ possesses a higher affinity than DNA pol β for Mg^2+^ that may be used as cofactor *in vivo* [76]. Further studies of morpholinonucleoside analogs both on DNA pol β and DNA pol λ could result in the development of potent and selective inhibitors of these DNA repair pols.

The classical approach to develop chemotherapeutic agents targeting DNA pols was to investigate sugar or nucleobase modiﬁcations in the nucleoside precursor, based on a SAR method [77,78,79,80]. A great advantage for the development of new effective NAs comes from the analysis of the crystal resolutions of DNA pols in complex with different NAs. As mentioned above, NAs used in the treatment of viral infections such as HIV, exert their adverse effects principally by inhibiting DNA pol γ, the mitochondrial replicase and main repair DNA pol. Modeling studies were conducted using *E. coli* DNA pol I and T7 DNA pol crystal structures to model DNA pol γ in complexes with different NRTIs [81]. These studies, together with mutagenesis and steady-state kinetic analysis, allowed the identification of three highly conserved amino acid residues in the active site of DNA pol γ, namely Tyr951, Tyr955, Glu895, that are responsible for the selection between dNTPs and NRTIs. In particular, Tyr955 partially accounts for the ability of DNA pol γ to incorporate stavudine (D4T) and carbovir, while Tyr951 results largely responsible for the incorporation of D4T as well as dideoxynucleotides. This kind of analysis was possible because DNA pol γ belongs to the family A DNA pols, which is represented by the well-studied *E. coli* DNA pol I and T7 DNA pol. The data obtained from these structures can be used to develop NAs showing higher selectivity towards nuclear DNA repair pols and lower affinity towards DNA pol γ. Such studies can now be improved by the recent crystal structure resolution of the DNA pol γ holoenzyme (the catalytic subunit Pol γA and the accessory subunit Pol γB, which increases enzyme processivity) [82]. Little structural information regarding DNA repair pols in complexes with therapeutically approved NAs is available. Recently, two crystal structures of DNA pol λ in complex with araC and dFdC were solved [74] (see above), while DNA pol β was co-crystallized together with ddC [83]. DNA pol β was shown to interact with the base moiety of ddC through the side chain of Asn279 and, more importantly, with the sugar moiety of ddCTP (C2' and C3') through van der Waals contacts between the protein backbone atoms of Tyr27, Phe272 and Gly27, that may participate in nucleotide selectivity. Surely, the lack of crystallographic resolutions of these DNA pols in complexes with other clinically approved NAs represents an obstacle for the design of new effective molecules. On the other hand, thanks to many crystallographic resolutions as well as pre-steady state kinetic and SAR analysis of DNA pol β and λ with natural or unnatural substrates [77,78,79,80,84,85,86,80,84], the mechanistic details governing substrate binding and catalysis are known for these two pols to a much deeper level than for the majority of other mammalian pols (with the exception maybe of the translesion enzymes DNA pols η, ι and κ). Such an improved understanding can provide useful information for the design of new effective NAs.

#### 3.1.2. Nucleoside Analogs Interactions with Other Intracellular Targets

In order to develop NAs useful in clinical treatment, it is important to design molecules not only potent and selective toward the target DNA pol, but also showing excellent pharmacokinetic properties. Indeed NAs are usually administered as nucleosides (prodrugs) and need to interact with several classes of enzymes in order to penetrate into the cells and be converted into their active triphosphate forms. Nucleoside analogs can penetrate the cell membrane through diffusion or by receptor mediate transport, through the activity of multiple active nucleoside transporters (NTs). Once into the cell, the nucleoside analogs must be converted into their monophosphate form by specific cellular kinases, mainly deoxycytidine kinase (dCK) and thymidine kinases (TK). Their further conversion to the active triphosphate form depends on the activity of less specific purine or pyrimidine nucleotide kinases. Moreover the stability of the NAs within the cell depends on the activity of deaminases, which deaminate the nucleobases cytidine, adenine and guanine, as well as 5' nucleotidases (NT5C), which target the monophosphate form of the NAs.

The complex pharmacological properties of the clinically approved drugs dFdC and araC, offer a clear example of the relevance of these interactions for the efficacy of NAs. Both of them are transported across the cell membrane by NTs, either sodium-dependent (concentrative) (CNT) or sodium-independent (equilibrative) (ENT) [87,88,89]. While araC can also penetrate into the cell in the absence of NTs by diffusion [90], dFdC uptake strongly depends on the expression of ENT1 and its downregulation correlates with resistance towards the NA [91,92]. AraC and dFdC conversion to their monophosphate forms relies on the activity of dCK, but dFdC is phosphorylated, even to a lesser extent, also by thymidine kinase 2 (TK2), a mitochondrial enzyme. This reaction is the rate limiting step for further phosphorylation to their active triphosphate form. It is important to underline that dCK deficiency has been frequently reported as the main mechanism of intrinsic resistance to dFdC as well as to araC, both *in vitro* and *in vivo* [89,93]. araC monophosphate (araCMP) can be dephosphorylated back to the nucleoside form by the enzyme 5´-nucleotidase (NT5C2). Differently, the main enzymes responsible for the conversion of dFdC monophosphate form back to its nucleoside form are the 5’-nucleotidase 1A (NT5C-1A) and 5’(3’)-deoxyribonucleotidase [94]. In particular NT5C-1A overexpression seems to be involved in resistance to this NA [95,96]. dFdC stability in cell is also strongly correlated to the expression of cytidine deaminase (CDA), while impaired CDA activity may results in increased toxicity [92,97,98]; indeed 90% of dFdC deaminase events occur by CDA activity while the remaining 10% is due to the action of deoxycytidylate deaminase (DCTD). Also AraC and araCMP, can both be converted into the inactive, araU and araUMP forms, by CDA and DCTD, respectively [89].

The cytotoxic effects of NAs are also influenced by their complex metabolic interactions with intracellular targets other than DNA pols. After dFdC incorporation into DNA, the subsequent incorporation of just one additional nucleotide leads to termination of chain elongation [99]. In this way the non-terminal position of dFdC in the DNA chain prevents its detection by DNA repair machinery [100,101]. dFdC can also be incorporated into RNA chains but the effect of this behavior on cell function remains unclear [102]. Moreover dFdCDP strongly inhibits ribonucleotide reductase (RR) [103,104], resulting in a decrease of competing deoxyribonucleotide pools necessary for DNA synthesis. This determines also an impairment of dNTPs pools resulting in high intracellular concentrations of dFdC metabolites compared with the natural dNTPs, increasing the probability of successful incorporation of dFdC into nucleic acids. Many others NAs like araC, fludarabine, cladribine and clofarabine share the ability to inhibit RR. A mechanism of resistance towards NAs consists in an increased cellular concentration of the competing canonical dNTPs that, in turn, compete with the NA for DNA incorporation. This mechanism is well documented in the case of araC [105]; the concomitant administration of nucleoside analogs inhibiting RR, like fludarabine and cladribine, has been shown to increase the cellular ratio of araCTP/dCTP, increasing its toxic profile [106,107,108,109]. The same effect can be achieved using specific RR inhibitors like triapine [110] and could result in a general method to improve the efficacy of NAs in therapy. Moreover, since in transformed cells the dNTPs pools can be unbalanced [111], the possibility to choose between purine and pyrimidine NAs depending on the best NA/dNTP ratio, could also result in a higher efficacy of the therapy, representing another boost to the development of new NAs. Altogether these findings reveal the complexity of interactions that take place between a NA and the cell metabolism and that must be taken into account for the development of effective NAs.

### 3.2. Mono-, Di- and Triphosphate Analogs as Drugs

As discussed above, the existing NAs employed in therapy are administered as nucleosides and depend on *in vivo* phosphorylation to exert their effects in the form of nucleoside 5'-triphosphates. The phosphorylation of the nucleoside moiety is often the step limiting the intracellular concentration of the active triphosphate drugs and thus the efficacy of NAs. However, up to date, a number of important mononucleotide cancer and antiviral drugs (cidofovir, tenofovir disoproxil fumarate, adefovir dipivoxyl) have also reached the clinical phase [112]. These kinds of drugs generally present a modified nucleoside monophosphonate that can bypass the initial activating phosphorylation step. Such residues mimic an α-phosphate of a natural nucleoside monophosphate (NMP), presenting a stable C–P link joining the nucleoside to the phosphonophosphate moiety and thus resulting in chemical and metabolic stability that is not available to α-phosphate derivatives. 

The negative charges carried by the phosphate moiety represent the most important barrier for the use of mononucleotide analogues in therapy, since they determine their low oral bioavailability and poor penetration into cells. Anyway, prodrug strategies can be elaborated in order to circumvent this problem and some antiviral nucleoside monophosphonates in orally administrable form have already been developed [112,113,114]. The increasing understanding of cellular drug transporters biology, will improve the pharmacological models allowing the possibility to rationalize the design of effective monophosphate drugs.

If the administration of monophosphate NAs greatly improves the efficacy of these drugs, the possibility to employ derivatives of di- and triphosphate nucleotide drugs will completely avoid the requirement for *in vivo* phosphorylation, resulting in an even greater advantage, opening the way to new chemotherapies for DNA replication-related diseases. Up to date there are no di- or triphosphate drugs in clinical trial, but some strategies to design such kind of drugs exist and they are based on chemical modifications in the phosphorus groups of the dNTP [115].

#### 3.2.1. dNTPs Modiﬁed at the γ-Phosphorus

**Figure 3:**
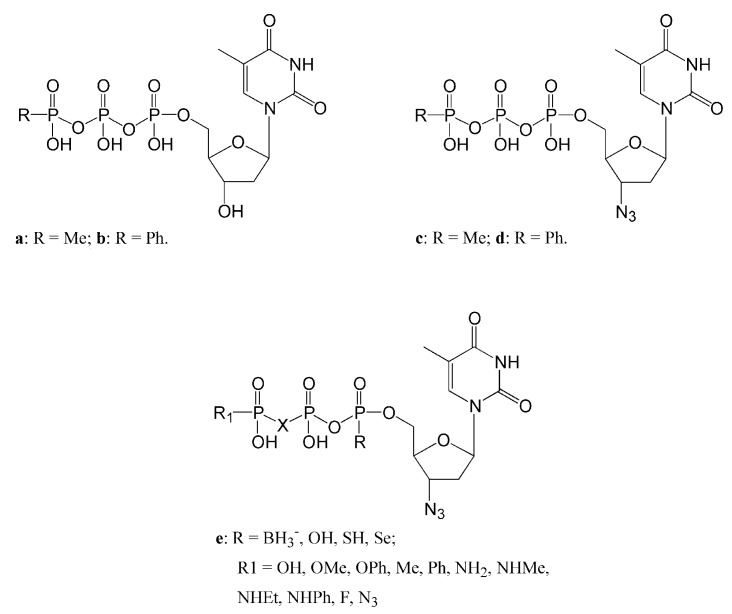
Structures of nucleoside triphosphate analogs modiﬁed at the β and/or γ-phosphate.

Modifications at the γ-phosphorus of a dNTP should result in a fewer steric constraints than changes within the β or α-phosphorus. In particular, the presence of a hydrophobic group at the γ-phosphorus (Figure 3) was shown to increase the selectivity of 3-azido-TTP analogues toward HIV-1 RT with respect to DNA pol α and β, determining a reduction of the adverse effect of these drugs. Moreover, these γ-phosphorus modifications confer a longer half life in serum (10-fold longer compared to the parent compounds) suggesting that this approach might result in the realization of more effective triphosphate drugs [116].

#### 3.2.2. Dinucleoside Tetraphosphates

**Figure 4:**
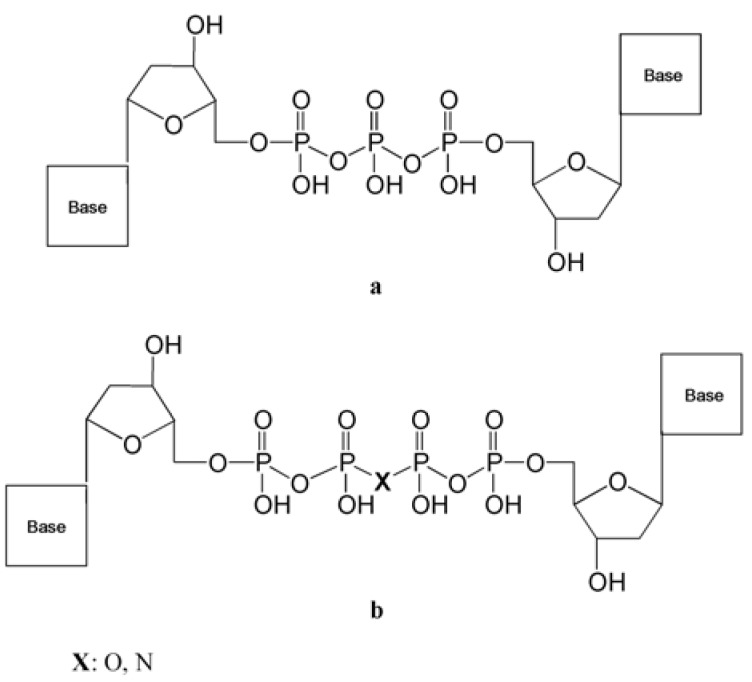
Structures of dinucleoside tri- and tetraphosphates analogs.

HIV-1 RT is able to remove the last incorporated nucleotide (or nucleotide analog such as AZT or d4T) on a growing DNA strand by using an ATP or GTP molecule with the formation of a dinucleoside tetraphosphate. Further modiﬁcations of dinucleoside tetraphosphates (Figure 4) synthesized by linking a dideoxynucleoside monophosphate (ddNMP) moiety to a nucleoside triphosphate (NTP) were investigated with the aim to create specific inhibitors of the enzyme [117]. 

Such compounds exhibited up to 120-fold higher inhibition potencies toward thymidine analogue resistant mutants of RT than toward the wt enzyme, suggesting a possible role for these drugs to delay or prevent the selection of mutations conferring enhanced primer unblocking activity to RT. Other than HIV-1 RT, dinucleoside tri- and tetraphosphates were shown to inhibit the bacterial DNA pols Taq and pol I from *E. coli* as well as human DNA pols α and β [118], underlining their possible use as effective inhibitors of these enzymes.

#### 3.2.3. dNTPs Modiﬁed at the α-Phosphorus and β,γ Bridging Analogs

Replacement of a non-bridging oxygen atom of the α-phosphate group with a borane moiety (Figure 3) has been show to greatly improve the catalytic rate of purine analog incorporation (70- and 13-fold with respect to AZT and ddATP) by RT mutation like Q151M, that are known to provide resistance towards these drugs decreasing their k_pol_. However such compounds, despite exhibiting a more lipophilic character if compared with the canonical triphosphate analogs, cannot be used as prodrugs due to lack of stability and poor cellular permeability. To overcome this problem, strategies involving modifications at the level of β,γ-bridging have been proposed. Replacement of the β,γ-bridging oxygen with a methylene, a halomethylene, or an imido group, were all shown to increase the half-lives of triphosphate analogs in serum. In particular, analogues containing a β,γ-diﬂuoromethylene or -dichloromethylene, exhibited a half life in serum of more than 48 hours, 4-fold and 24-fold longer than AZT-5-α-P-boranoTP (6 h) and AZTTP and TTP (2 h), respectively [119]. Double substitution at non-bridging oxygen atoms of the α-phosphate group and at β,γ-bridging level could result in more effective NAs inhibitors showing better pharmacokinetics properties. As an example, replacement of the β,γ-bridging oxygen with β,γ-diﬂuoromethylene combined with the substitution of a non-bridging oxygen of the α-phosphate group with a sulfur or selenium atom in the molecules of dideoxynucleotides, lead to a 20-fold increase in the inhibition potencies compared to the corresponding triphosphate [119]. In conclusion, modifications at the level of the phosphorous groups in the molecules of triphosphate NAs should allow the identification of more effective and selective DNA pols inhibitors, showing pharmacokinetic properties suitable for clinical trial studies.

### 3.3. Nucleotide Analogs Targeting Specific DNA Lesions

Conventional cancer therapy is based, among other approaches, on the administration of alkylating agents. These drugs modify chemical groups in the DNA helix, thus introducing DNA damage. Tumor cells have lost the ability to block their cell cycle in an attempt to repair such lesions, so DNA duplication results in collapsed replication forks and/or double strand breaks that, in turn, cause cell death. However, prolonged administration of such compounds can lead to the acquisition of resistance to the therapy. This can be due to different mechanisms; one of these consists in the enhancement of the activity of DNA repair pathway like Base Excision Repair (BER) [120]. In the initial step of BER, the damaged base is excised by the activity of damage specific glycosylases with the concomitant formation of an abasic site (AP). The following reactions in the BER pathway require the incision of the sugar-phosphate chain by APE1 and the subsequent recruitment of repair DNA pol (pol β or pol λ) that fill the DNA gap intermediate and, finally, Ligase I seals the DNA chain.

Under chemotherapy regimens with alkylating drugs, even if the BER pathway is fully active, the cell cycle progression is forced in the cancer cell, due to mutations in the checkpoint pathways, preventing the correct execution of the BER reactions. As a consequence, some BER intermediates like AP sites can be present when the DNA replication occurs. The availability of chain terminators NAs that can be incorporated only opposite AP sites could result in an enhancement of the toxicity of alkylating agents used for chemotherapy, specifically in cancer cells. In the same way, NAs able to efficiently pair and be incorporated only opposite alkylated bases, blocking the progression of DNA synthesis, should result in an improvement of the therapy.

**Figure 5:**
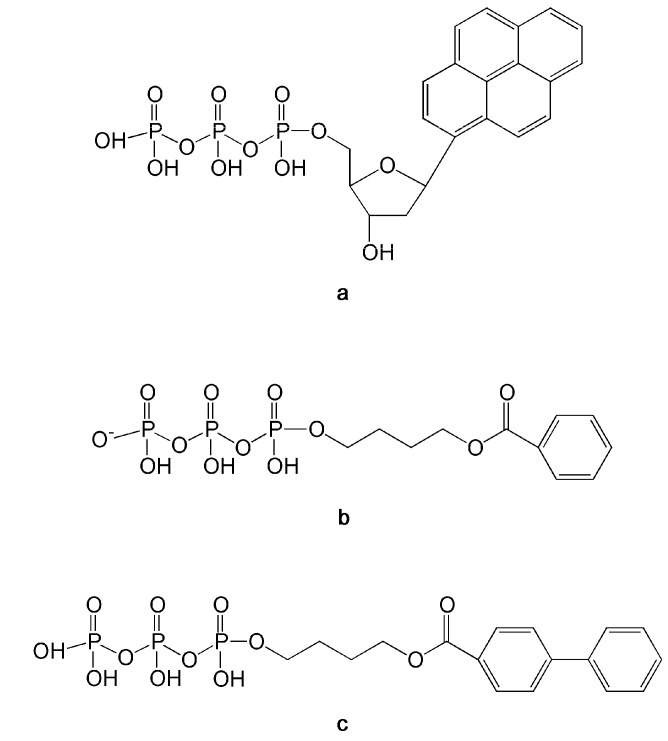
Structures of nucleoside and non-nucleoside triphosphate analogs efficiently pairing with AP site. (**a**) Pyrene nucleotide (dPTP); (**b**) 4-O-benzoyloxylbutyl triphosphate; (**c**) (biphenylcarboxyl)-4-oxybutyl triphosphate.

Compounds that can be uniquely incorporated opposite an AP site by specific DNA pols have been already developed. An important example is represented by the substitution of the nucleobase with a pyrene moiety (Figure 5a) [121]. The resulting pyrene nucleotide (dPTP) can be incorporated by a Klenow fragment (Kf) opposite an AP site more efficiently than canonical nucleotides. Moreover Kf is not able to elongate dPTP after its incorporation opposite an AP site. Other molecules can be incorporated only opposite an AP site by BER enzymes like pol β and pol λ [122,123]. These compounds are non-nucleoside alkyltriphosphate analogues like 4-O-benzoyloxylbutyl triphosphate and (biphenylcarboxyl)-4-oxybutyl triphosphate (Figure 5b and Figure 5c) that lack both the sugar and the nucleobase moieties. For these reasons, such compounds cannot be substrates of the cellular kinases that convert nucleosides in their triphosphate active forms. Since triphosphate molecules show poor membrane permeability and a short half life in serum, these alkyl triphosphates are not suitable for clinical treatment. Nevertheless, they have provided useful indications on the large flexibility of DNA pol λ active site, which was able to incorporate the compound (biphenylcarboxyl)-4-oxybutyl triphosphate opposite an AP site with an efficiency only 2.2-fold higher than canonical nucleotides dATP, demonstrating the possibility to design NAs exhibiting such behavior. The synthesis of such NAs or others able to be efficiently incorporated opposite other damaged nucleobases, is an intriguing option to improve the efficacy of alkylating chemotherapy.

### 3.4. Strategies to Deliver Mono-, Di- and Triphosphate Analogs

A strategy to overcome the problems related to the administration of phosphorylated NAs, like poor membrane permeability and rapid degradation in plasma consists of using prodrugs of the phosphorylated metabolites. As a result of their high lipophilicity, masked nucleotides are able to penetrate cell membranes in their intact form and are therefore not prone to degradation by nonspecific plasma phosphatases. Prodrugs for nucleoside monophosphates (NMPs) have been designed employing masking moiety like cycloSal, phosphoramidate, bis(sacylthioethyl) and bis(pivaloxymethyl) [124,125,126]. An approach to realize diphosphate prodrugs consists to attach different acyl moieties to the β phosphate of the analogs [127,128]. In this way the mixed anhydride bond is cleaved faster than the phosphate anhydride bond. This was proven in hydrolysis studies in an aqueous buffer, but undesired decomposition occurred in biological media like RPMI culture medium [127]. More recently, a diphosphate prodrug was created by using small-molecule masking tactics [129,130] employing a bis(4-acyloxybenzyl)- moiety as masking unit. Other promising methods for the delivery of triphosphates analogs relied on nanogel carrier composed of amphiphilic polymers and cationic polyethylenimine. Administration of AZTTP in a nanogel formulation towards two breast cancer cell lines, MCF7 and MDA-MB-231, showed enhanced cytotoxicity, exhibiting IC_50_ values 130- to 200-times lower than for AZT alone [131]. Other nucleotide triphosphate analogs like cytarabine (araC), gemcitabine (dFdC), and ﬂoxuridine (FdU), once encapsulated in biodegradable PEG-cl-PEI or F127-cl-PEI nanogel, exhibited equal or higher activity than the parent nucleoside drug in breast and colorectal cancer cell lines. Moreover, in animal models, nanoformulations exhibited higher activities than nucleoside analogue drugs [132].

## 4. Conclusions

It is not too far fetched to draw a parallel between viral-infected cells and cancer cells. Both kinds of cells are sick. They show alterations in several key regulators of cell cycle, cell proliferation and DNA damage response proteins. While in infected cells these alterations are caused by the action of viral proteins, in cancer cells they are due to mutations. Nonetheless, the final outcome is similar: both infected and cancer cells have lost their homeostatic equilibrium. Moreover, just as the virus is relying on a limited number of key interactions with cellular proteins to efficiently replicate, cancer cells often rely on a narrow range of critical pathways to survive avoiding apoptosis. Finally, as much as viruses develop resistance to drugs, also cancer cells can become resistant to chemotherapy. Given these similarities, it is not surprising that scientists have started to realize that the conceptual framework successfully developed to design chemotherapeutic strategies against viral infections, can also prove to be valid for cancer chemotherapy. Similar issues such as molecular targeted therapies, drug resistance, combinatorial chemotherapy, are becoming more and more important in the cancer field. Along this line, targeting viral replication enzymes has proven a very successful approach for viral infections as diverse as hepatitis and AIDS and the knowledge gained in designing specific viral DNA pols inhibitors is now available for the development of inhibitors of cellular DNA pols. Thus, a time has come when the ability of a small molecule to inhibit cellular DNA pols is not regarded anymore as a potential drawback, but as an interesting property.
